# Monoclonal Antibodies Targeting CGRP: A Novel Treatment in Vestibular Migraine

**DOI:** 10.3390/medicina59091560

**Published:** 2023-08-28

**Authors:** Andrea Lovato, Caterina Disco, Andrea Frosolini, Daniele Monzani, Francesco Perini

**Affiliations:** 1 Otorhinolaryngology Unit, Department of Surgical Specialties, Vicenza Civil Hospital, 36100 Vicenza, Italy; 2 Otorhinolaryngology Unit, Department of Surgical Specialties, San Gaetano Clinic, 36016 Thiene, Italy; 3 Neurology Unit, Department of Neuroscience, Vicenza Civil Hospital, 36100 Vicenza, Italy; 4 Maxillofacial Surgery Unit, Department of Medical Biotechnologies, University of Siena, 53100 Siena, Italy; 5 Otorhinolaryngology Unit, Department of Surgical Specialties, University of Verona, 37100 Verona, Italy

**Keywords:** vestibular migraine, migraine, dizziness, monoclonal antibodies, nystagmus, vertigo

## Abstract

*Background*. Monoclonal antibodies (mAbs) directed against the calcitonin gene-related peptide (CGRP) or its receptor represented the first targeted and specialized approach to migraine prophylaxis. Nevertheless, they have been rarely considered in the treatment of vestibular migraine (VM). Our aim was to evaluate the effectiveness of anti-CGRP mAbs in VM patients who did not respond to conventional migraine treatments. *Methods*. Consecutive VM patients treated with erenumab were considered. As a comparison, we considered the same VM patients during conventional migraine treatments (i.e., propranolol, flunarizine, or valproic acid), which were tried before mAbs therapy. Videonystagmography, the Italian version of the Dizziness Handicap Inventory (DHI) questionnaire, and migraine days over the last 3 months were evaluated in all patients before and after treatments. *Results*. In the present retrospective study, we included 21 female and 2 male VM patients, mean age 45.2 years. All patients underwent contrast-enhanced magnetic resonance imaging that ruled out other causes of vertigo. The DHI questionnaire significantly improved after mAb therapy (*p* < 0.0001). Mean migraine days over the last 3 months were significantly reduced after treatment (*p* = 0.001). Videonystagmography was altered in 11 (48%) patients prior to monoclonal antibodies. We found vertical positional nystagmus in 9 patients and horizontal positional nystagmus in 2 patients. After the treatment, we found vertical positional nystagmus only in 1 patient (*p* = 0.002). When patients were treated with conventional therapies, there was no significant reduction in DHI, and instrumental vestibular examinations remained altered. *Conclusions*. VM patients using anti-CGRP mAbs experienced a reduction in the dizziness-derived handicap, as reported in the DHI questionnaire. Furthermore, these treatments were significantly associated with a normalization of vestibular instrumental analysis. These findings were not seen with conventional treatments. Treatment with anti-CGRP mAbs may be effective in VM patients who did not respond to conventional migraine treatments. These findings should be tested in large, randomized clinical trials.

## 1. Introduction

### 1.1. Vestibular Migraine: Current Definition, Diagnosis and Treatment

Vestibular migraine (VM) represents a type of migraine characterized by vestibular symptoms, encompassing spontaneous vertigo, positional vertigo, vertigo triggered by visual stimuli, and vertigo provoked by head motion. Coined in 1999, the term “vestibular migraine” was introduced to categorize individuals experiencing episodic vertigo associated with migraines [1]. Subsequently, in 2012, the International Classification of Headache Disorders and the Barany Society joined forces to establish the following diagnostic criteria for VM: (i) At least five episodes that involve (ii) a history of migraine with or without aura based on the International Classification of Headache Disorders, along with (iii) moderate to severe vestibular symptoms lasting 5 min to 72 h, including spontaneous vertigo attacks, positional vertigo triggered by head position, visually induced vertigo, and vertigo provoked by head motion. Additionally, (iv) a minimum of 50% of episodes must be accompanied by at least one migraine headache feature such as throbbing headache, phonophobia, or photophobia. Lastly (v), the diagnosis should not be better explained by another headache disorder or vestibular condition. These criteria guide the identification of VM through a comprehensive evaluation of clinical history and symptoms, allowing differentiation from other disorders [2]. VM presents a spectrum of vertigo manifestations, encompassing spontaneous, positional, visually induced, and head motion-induced vertigo, along with head motion-induced dizziness accompanied by nausea. Some patients exhibit a progression from spontaneous vertigo to positional or head motion-induced vertigo over hours or days. The persistence of positional vertigo hinges on specific head positions [2]. Auditory symptoms such as hearing loss, tinnitus, and aural pressure occur during acute attacks in 20% to 40% of VM patients. Hearing loss is generally transient and mild, with minimal progression. Approximately 20% of individuals develop mild bilateral downsloping sensorineural hearing loss over time [2]. VM’s prevalence ranges from 1% to 2.7%, establishing it as a prominent neurological cause of adult vertigo [3]. The occurrence of migraines among patients with recurrent unexplained vertigo hovers between 60% and 80% [3]. The major differential diagnosis regards Ménière’s disease (MD): In precedent studies, more than half of VM patients were initially misdiagnosed with MD; in other cases, approximately 25% of subjects had a combination of Ménière’s disease and vestibular migraine (MDVM) [4]. Distinguishing between the two entities is challenging due to shared clinical features. A comprehensive diagnostic approach is crucial—combining core symptom assessment, audiological evaluation, vestibular function tests, and imaging for detecting the characteristic MD’s endolymphatic hydrops—for accurate differentiation and effective management. 

Unfortunately, there is a marked scarcity of randomized controlled trials in the acute management of VM, primarily focusing on triptans [5]. Correspondingly, few investigations have been conducted into VM prophylaxis, characterized by an overall dearth of high-quality evidence [5]. The following molecules have been mainly considered: Metoprolol, propranolol, venlafaxine, amitriptyline, flunarizine, topiramate, or valproic acid [5]. No clear recommendations for the prescription of such therapies have been made [6].

### 1.2. The Calcitonin Gene Related Peptide (CGRP) 

CGRP, a neuropeptide comprising 37 amino acids, was first discovered three decades ago. Its synthesis arises from alternative RNA processing of the calcitonin gene, resulting in two primary forms (α and β) [7]. This peptide belongs to a group of molecules that interact with a distinct receptor family consisting of the calcitonin receptor-like receptor (CLR) coupled with an essential receptor activity modifying protein (RAMP) essential for complete functionality [7]. CGRP functions as a potent vasodilator, and its properties extend to protective mechanisms vital for physiological and pathological contexts involving the cardiovascular system and wound healing. Mainly released from sensory nerves, CGRP also features in pain pathways [7]. Within the realm of migraine, CGRP is now established as a pivotal participant. While the understanding of VM’s pathophysiology remains incomplete, it shares resemblances with migraine, often co-occurring with a long-standing history of migraine in patients [8]. Neuroanatomical connections between the vestibular system and nociceptive brainstem regions are apparent, with heightened signal transmission between these systems in VM patients [9]. Migraine symptoms appear linked to changes in ion channel function, leading to altered neural activity in the trigeminovascular system [10], culminating in the release of neurotransmitters such as substance P and CGRP [11]. Moreover, CGRP receptors are expressed in the vestibular system and play a role in motion sickness [12,13]. A recent study by Tian et al. [14] used intermittent nitroglycerin administration to induce a rat model of chronic migraine and vestibular dysfunction. The study observed elevated expression of CGRP, as well as its receptor components CLR and RAMP1, in the vestibular nucleus (VN). The researchers used a CGRP1 receptor antagonist; inhibiting the CGRP1 receptor attenuated mechanical allodynia, thermal hyperalgesia, and vestibular dysfunction. This was associated with downregulation of synaptic-associated proteins, restoration of synaptic ultrastructure, and suppression of neuronal activation in the vestibular nucleus. In fact, the PKC/ERK/CREB signaling pathway played a role in the CGRP-mediated regulation of synaptic transmission, and inhibiting the CGRP1 receptor reduced the expression of synaptic proteins and downstream phosphorylation of ERK and CREB. This signaling pathway may contribute to the maintenance of central sensitization and synaptic transmission efficiency. The study examined the dendritic synapses and their ultrastructure: There was an increase in dendritic spine density, which was normalized by treatment. Levels of c-Fos in VN, a marker of neuronal activation after noxious stimulus, were reduced by CGRP1 receptor inhibition [15]. All these findings suggested that CGRP1 receptor antagonism might alleviate neuronal hyperactivity and maladaptivity associated with vestibular dysfunction by various mechanisms. Moreover, a recent study published in a preprint suggested more pathogenic roles for the CGRP pathway. Rahman et al. [16], by utilizing behavioral tests such as the elevated plus maze and the rotarod assay, revealed that CGRP administration, particularly when coupled with a vestibular challenge (such as off-vertical axis rotation), can induce anxiety-like behaviors and affect balance function in mice. These dysfunctions are particularly manifested as an increased preference for enclosed spaces in the test. Importantly, these effects are more pronounced in female mice, highlighting a gender-based disparity in CGRP-induced anxiety and motion-related behaviors. This study revealed the potential involvement of CGRP in motion-induced anxiety and dynamic balance and suggests that targeting CGRP pathways could have therapeutic implications for these symptoms.

### 1.3. Monoclonal Antibodies (mAbs) Targeting the CGRP in Migraine

Since 2018, monoclonal antibodies (mAbs) targeting the calcitonin gene-related peptide or its receptor have been utilized for preventing both episodic and chronic migraines, signifying a pioneering approach in specific and selective migraine prophylaxis [8]. These mAbs function by interfering with CGRP-associated signaling pathways [7]. Despite variations in attributes such as composition (fully human, humanized), target (CGRP, CGRP receptor), delivery (subcutaneous, intravenous), and frequency (monthly, quarterly), anti-CGRP mAbs exhibit a strikingly uniform clinical profile. Demonstrating effectiveness and favorable tolerance, these mAbs prove beneficial for individuals experiencing episodic or chronic migraines, even in cases involving medication overuse or prior treatment failures (ineffective efficacy or tolerability in 2 to 4 preventive treatments) [17,18]. Novel anti-CGRP mAb therapies have exhibited notable proportions of responders and impressive efficacy-to-tolerability ratios in migraine patients, marking a significant advancement beyond conventional standards of care [19,20,21]. This novel class of migraine preventive treatments has focused on improving patients’ quality of life (QoL) and reducing migraine-related disability. Various patient-reported outcome (PRO) measures, such as the Headache Impact Test (HIT-6), Migraine Disability Assessment Questionnaire (MIDAS), Migraine-Specific Quality of Life Questionnaire (MSQ), and others, have been employed to assess the impact of these antibodies on migraine sufferers. Clinical trials of CGRP-mAbs have consistently demonstrated their efficacy in decreasing migraine frequency, severity, and associated disability, thereby enhancing patients’ functional capacity and overall well-being. The PRO data underscore the potential of CGRP-mAbs to address the unmet needs of migraine patients by significantly improving their QoL and reducing the burdensome effects of this neurological disorder [22]. In this context, the potential of CGRP inhibitors in treating VM holds captivating promise, albeit only a single retrospective study has reported the possible efficacy of anti-CGRP mAbs in VM prevention [23].

### 1.4. Objective of the Study

In this retrospective study, we aimed to assess with objective measures the efficacy of anti-CGRP mAbs in VM patients who displayed unresponsiveness to conventional migraine therapies.

## 2. Materials and Methods

### 2.1. Study Design

The research adhered to the principles outlined in the Helsinki Declaration. As a retrospective investigation, the Ethics Committee of Vicenza Hospital exempted the need for formal approval. The analysis of the data followed compliance with Italian privacy regulations and sensitive data laws. In this retrospective crossover study, we scrutinized the medical records of successive VM patients who underwent treatment at both Vicenza Civil Hospital and San Gaetano Clinic. The enrollment period spanned from 1 January 2021 to 1 January 2023.

### 2.2. Inclusion and Exclusion Criteria

Participants were enrolled in the study based on the following criteria: (i) Age exceeding 18 years; (ii) a VM diagnosis conforming to the criteria established by the International Classification of Headache Disorders and the Barany Society [24]; (iii) migraine patients being prescribed anti-CGRP mAbs in accordance with criteria mandated by the Italian Medicine Agency (adult patients encountering ≥ 8 monthly migraine days within the past 3 months, coupled with documented unsuccessful attempts with >3 migraine preventive medication classes, encompassing beta-blockers, anticonvulsants, and tricyclics) [21]. Exclusion criteria encompassed: (i) A diagnosis of benign paroxysmal positional vertigo; (ii) a diagnosis of Meniere’s disease; (iii) an identification of other neurological disorders; and (iv) co-occurring psychiatric conditions. As a comparison, we considered the same patients during conventional migraine treatments (i.e., beta-blockers, anticonvulsants, and tricyclics), which were tried before anti-CGRP mAbs. These therapies were stopped during anti-CGRP mAbs.

### 2.3. Outcomes

Migraine days over the last 3 months were asked of all patients before and after treatments. 

All assessments of vestibular function, both prior to and post-conventional or mAbs therapy, were pivotal. Our standard practice entails employing videonystagmography for evaluating equilibrium disorders. Assessments were not conducted during instances of acute vertigo attacks in VM. Presently, videonystagmography stands as the prevailing method for tracking eye movements, utilizing infrared light. The examination protocol incorporates spontaneous and positional testing as well as caloric testing, which collectively consumes approximately thirty minutes. To ensure accurate results, patients should abstain from sedatives and vestibular suppressant medications for 48 h prior to testing. Spontaneous nystagmus and gaze-evoked nystagmus are captured without fixation (under conditions of darkness), with patients directed to gaze 30 degrees to both the right and left. Positional testing comprises static and paroxysmal (dynamic) testing. During static positional assessments, patients are sequentially positioned in the sitting, supine, head left, and head right positions, all in darkness. Paroxysmal positional testing employs the Dix–Hallpike maneuver, entailing a sequence of positions transitioning from sitting with the head straight, to sitting with a 45-degree head turn, followed by reclining with the head still turned and the neck extended at a 20-degree angle below horizontal. Subsequently, the patient is returned to an upright seated position, and the maneuver is repeated with the head turning in the opposite direction. 

Every patient completed the Italian version of the Dizziness Handicap Inventory (DHI) questionnaire before and after receiving either conventional or mAb treatments [25]. Comprising 25 items, the DHI is a self-report questionnaire that gauges the extent of dizziness’s impact on daily life through self-assessed handicaps. It assesses three domains: (i) Functional (9 questions, 36 points); (ii) emotional (9 questions, 36 points); and (iii) physical (7 questions, 28 points). Item scores are aggregated, yielding a total score ranging from 0 to 100. Higher scores denote a greater perceived handicap stemming from dizziness.

### 2.4. Statistical Analysis

The study employed statistical tests to analyze treatment effectiveness. These included the Fisher exact test, Mann–Whitney U test, and chi-square test, each chosen based on data type and research goals. A significance level of *p* < 0.05 was set to determine statistical significance. The Social Sciences version 17 statistical package (SPSS Inc., Chicago, IL, USA) was used for all analyses.

## 3. Results

In the present study, we included 23 VM patients with a mean age of 45.2 years (standard deviation [SD] 7.8 years). Clinical and demographic characteristics of the considered subjects are reported in Table 1. All patients underwent contrast-enhanced magnetic resonance imaging that ruled out other causes of vertigo. 

Patients received subcutaneous erenumab (140 mg) every 28 days, and the mean duration of follow-up was 26.4 weeks (SD 2.1 weeks). Before anti-CGRP mAbs, 3 patients complained of spontaneous acute vertigo attacks, 10 of positional vertigo, 4 of head motion-induced dizziness/vertigo, and 6 of postural unsteadiness. Vestibular symptoms significantly improved after mAbs therapy; we found a reduction in the mean DHI questionnaire from 30.2 (SD 7.2) to 8.1 (SD 3.1) (Mann–Whitney U test; *p* < 0.0001). Moreover, mean migraine days over the last 3 months were significantly reduced after treatment (Fisher exact test; *p* = 0.001) from a mean of 12.4 days to a mean of 5.1 days. Videonystagmography was altered in 11 (48%) patients prior to monoclonal antibodies. We found vertical positional nystagmus in 9 patients and horizontal positional nystagmus in 2 patients. After the treatment, we found vertical positional nystagmus only in 1 patient (chi-square test; *p* = 0.002). No side effects were observed during the study period.

As a comparison, we considered the same 23 patients during conventional migraine treatments, which were tried before mAb therapy. The mean duration of follow-up before interrupting conventional therapies was 28.3 weeks (SD 3.1 weeks). Follow-up was not different between the two groups. Twelve patients were using propranolol (40 to 80 mg), 7 were using flunarizine (5 mg), and 4 were using valproic acid (500 to 800 mg). At first evaluation, the mean DHI was 33.5 (SD 6.9), and videonystagmography was altered in 10 patients. At the follow-up visit (after a mean of 28.3 weeks), the mean DHI was 31.8 (Mann–Whitney U test; *p* = 0.2), and instrumental vestibular examination remained altered in 10 patients (chi-square test; *p* = 1.0). Mean migraine days over the last 3 months were 13.2 days before and 12.4 days after treatment (Fisher exact test; *p* = 0.2).

## 4. Discussion

In this retrospective crossover study, we assessed the efficacy of anti-CGRP mAbs in VM patients who had not responded to traditional migraine treatments. Among the 23 participants included in our study, pre- and post-treatment DHI demonstrated a significant reduction in scores following anti-CGRP mAb treatment (from 30.2 ± 7.2 to 8.1 ± 3.1; *p* < 0.0001), signifying an amelioration in dizziness-related quality of life. This reduction implies a decreased impact of dizziness on physical, emotional, and functional aspects of participants’ lives. The DHI proves valuable, consistent, and valid in gauging self-assessed impairment linked to dizziness. It has been validated in cases of acute [25] and recurrent [26] vestibular disorders, as well as chronic dizziness [27]. Elevated DHI scores have been documented in VM patients [28], and the questionnaire has been employed to examine the effects of personalized vestibular rehabilitation (VR) programs on balance, gait performance, and self-perceived handicap in VM patient groups [29]. Moreover, the objective testing performed in our study (i.e., videonystagmography findings) significantly improved after anti-CGRP mAbs, with pathological signs dropping from 48% pre-treatment to 4% after-treatment (*p* = 0.002). A recent study by Fu et al. [30] comprehensively evaluated vestibular and oculomotor function in individuals diagnosed with VM: A cohort of 41 VM patients underwent a battery of vestibular assessments, including cervical and ocular vestibular evoked myogenic potential (cVEMP, oVEMP), video head impulse test (vHIT), caloric test, and videonystagmography. The findings revealed that 73% of VM patients exhibited abnormal vestibular function test results, with varying prevalence observed in different tests. Our lower proportion (48%) can be interpreted as indicating that we adopted fewer objective tests (i.e., videonystagmography) than the other research group. 

As a comparison for our results, we considered the same 23 patients during conventional migraine treatments (i.e., propranolol, flunarizine, or valproic acid), which were tried before mAbs therapy. Patients had no significant reduction in DHI, and instrumental vestibular examination remained altered (from 33.5 ± 6.9 to 31.8). As previously reported, conventional migraine prophylactic treatments have proven scarce effectiveness in VM treatment [5,6]. In a study by Salviz et al. [31], sixty-four subjects with confirmed VM were randomly assigned to receive either propranolol (group P, *n* = 33) or venlafaxine (group V, *n* = 31) for VM prophylaxis. After 4 months of treatment, both groups exhibited significant reductions in DHI total scores (from 55.8 ± 2.7 to 31.3 ± 3.7 for the P group and from 50.9 ± 2.5 to 19.9 ± 2.9 for the V group), vertigo symptom scale (VSS), and vertigo attack frequency (*p* < 0.001 for all), with similar treatment effects in both groups (*p* > 0.05). Notably, both venlafaxine and propranolol failed to completely resolve the handicap related to VM, as the DHI remained in both cases above 16 out of 100, therefore indicating a mild handicap. 

To the best of our knowledge, this was the first study that included instrumental analysis prior to and after mAb therapy in VM patients. Russo et al. [32] performed a prospective observational cohort study in order to evaluate the efficacy of erenumab, fremanezumab, and galcanezumab for the treatment of 50 vestibular migraine patients. They found an excellent response to treatments, as 45 (90%) patients had at least a 50% reduction in vertigo frequency, and mean monthly days with dizziness/vestibular symptoms showed an overall significant decrease [32]. Hoskin et al. [23] assessed the efficacy of four different anti-CGRP mAbs (erenumab, galcanezumab, ubrogepant, and fremanezumab) in alleviating VM symptoms. Their retrospective monocentric study was conducted between 2016 and 2020: Out of 28 patients, 25 were followed up, and 21 demonstrated varying levels of improvement in VM symptoms, with 15 showing moderate to significant improvement with a consistent trend across the different CGRP medications (according to a subjective report of symptom). Notably, this study lacked appropriate diagnostic methods and outcome measures for VM. 

Anti-CGRP mAbs have exhibited favorable tolerability profiles in clinical trials, although individual responses can vary. Possible adverse effects encompass local injection site reactions, infrequent hypersensitivity reactions that could manifest as rash, pruritus, or even anaphylaxis, as well as gastrointestinal symptoms such as nausea and diarrhea. The paradoxical occurrence of headaches post-administration, resembling flu-like symptoms including fatigue and muscle discomfort, and sporadic elevations in liver enzymes have also been documented. While concerns regarding cardiovascular effects are theoretically present due to CGRP’s impact on vascular function, clinical trials and precedent studies have not substantiated significant issues in this regard [23,32]. It is essential to recognize that adverse events are generally infrequent, with most patients tolerating these mAbs well; accordingly, no side effects were reported among the 24 patients treated in the present study.

In our patients, anti-CGRP mAb prophylactic treatment significantly reduced headache and vestibular complaints. There have been only a few randomized controlled trials investigating prophylactic approaches for VM. The Prophylactic Treatment of VM with Metoprolol (PROVEMIG) trial aimed to assess the efficacy of metoprolol compared to a placebo among individuals with VM. Regrettably, the trial did not definitively demonstrate metoprolol’s superiority [33]. In another randomized study, flunarizine exhibited an improvement in the frequency and intensity of vertigo, although without comparable benefits for headaches [34]. A recent systematic review and meta-analysis conducted by Yannakis et al. [35] evaluated the effectiveness of prophylactic medications for managing VM. The review included both randomized and non-randomized controlled trials. Propranolol and venlafaxine were found to significantly improve VSS and DHI scores, with propranolol demonstrating the highest success rate of symptom control. Therefore, the authors suggest that propranolol should be considered the primary treatment option for VM, followed by venlafaxine, while other medications such as amitriptyline, flunarizine, and cinnarizine showed only potential improvement trends without reaching statistical significance [35]. On the contrary, a well-structured systematic review concluded that the lack of available trials to inform practical recommendations for the pharmacological prevention of VM represents a critical gap in the field [36]. According to other authors, despite the common use of several drugs for treating vestibular migraine, their efficacy lacks proper substantiation [37]. Consequently, a cautious approach is recommended in prophylactic treatment of VM, considering the balance between potential benefits and known side effects that necessitates an individual patient assessment [37]. 

Our study possesses several strengths: (i) A clear research objective of assessing the efficacy of anti-CGRP mAbs in patients unresponsive to conventional migraine therapies; (ii) rigorous inclusion and exclusion criteria; (iii) a combination of patient-reported outcomes (i.e., DHI) and objective vestibular assessments (i.e., videonystagmography), contributing to a comprehensive evaluation of treatment outcomes; (iv) a direct comparison between anti-CGRP mAbs and conventional migraine treatments within the same cohort, providing valuable insights into treatment effectiveness. Moreover, a number of limitations need to be reported: (i) The study’s retrospective and monocentric design presents constraints in controlling data collection and limits broader applicability; (ii) the relatively small sample size (23 participants) may compromise generalizability; (iii) selection bias may arise from studying patients previously unresponsive to traditional treatments; (iv) the lack of randomization and multivariate analysis could confound treatment effects. Overall, while offering significant and promising insights, our study underscores the need for prospective, controlled trials with larger cohorts to enhance treatment validation and comprehension of VM management. Within this framework, the delineation of precise predictors of treatment response within more substantial cohorts holds potential clinical relevance, facilitating the refinement of tailored therapeutic approaches for patients diagnosed with VM. Moreover, in future studies, it would be interesting to deeply evaluate with adjunctive objective measures the correlation between anti-CGRP mAb effectiveness and both migraine and vestibular symptoms.

## 5. Conclusions

According to our results, VM patients using anti-CGRP mAbs experienced a reduction in the handicap resulting from dizziness, as reported in the DHI questionnaire. Furthermore, these treatments were significantly associated with a normalization of vestibular instrumental analysis. These findings were not seen using conventional treatments. Treatment with anti-CGRP mAbs may be effective in VM patients who did not respond to conventional migraine therapies. These findings should be tested in large, randomized clinical trials.

## Figures and Tables

**Table 1:** Demographic and clinical characteristics of included vestibular migraine patients.

Characteristics	Total subjects = 23
Female	21 (91%)
Caucasian	23 (100%)
Unilateral headache	18 (78%)
Associated photophobia or phonophobia	21 (91%)
Aura	0 (0%)
Mean duration of vestibular symptoms	37.9 months (SD 12.3 months)

Abbreviations: SD (standard deviation).

## Data Availability

The data supporting reported results can be obtained by the corresponding author upon request.

## References

[1] Dieterich M., Brandt T. (1999). Episodic vertigo related to migraine (90 cases): Vestibular migraine?. Neurol. J..

[2] Lempert T., Olesen J., Furman J., Waterston J., Seemungal B., Carey J., Bisdorff A., Versino M., Evers S., Kheradmand A. (2012). Vestibular migraine: Diagnostic criteria. Vestib. J. Res..

[3] Formeister E.J., Rizk H.G., Kohn M.A., Sharon J.D. (2018). The epidemiology of vestibular migraine: A population-based survey study. Otol. Neurotol..

[4] Chen J.Y., Guo Z.Q., Wang J., Liu D., Tian E., Guo J.Q., Kong W.J., Zhang S.L. (2023). Vestibular migraine or Meniere’s disease: A diagnostic dilemma. J. Neurol..

[5] Neuhauser H., Radtke A., von Brevern M., Lempert T. (2003). Zolmitriptan for treatment of migrainous vertigo: A placebo-controlled trial. Neurology.

[6] Beh S.C. (2022). Vestibular Migraine. Curr. Neurol. Neurosci. Rep..

[7] Russell F.A., King R., Smillie S.J., Kodji X., Brain S.D. (2014). Calcitonin gene-related peptide: Physiology and pathophysiology. Physiol. Rev..

[8] Dieterich M., Obermann M., Celebisoy N. (2016). Vestibular migraine: The most frequent entity of episodic vertigo. Neurol. J..

[9] Furman J.M., Marcus D.A., Balaban C.D. (2013). Vestibular migraine: Clinical aspects and pathophysiology. Lancet Neurol..

[10] Eggers Z.S.D. (2021). Episodic spontaneous dizziness. Continuum..

[11] Baloh R.W. (2020). Vestibular migraine i: Mechanisms, diagnosis, and clinical features. Semin. Neurol..

[12] Xiaocheng W., Zhaohui S., Junhui X., Lei Z., Lining F., Zuoming Z. (2012). Expression of calcitonin gene-related peptide in efferent vestibular system and vestibular nucleus in rats with motion sickness. PLoS ONE.

[13] Zhang Y., Zhang Y., Tian K., Wang Y., Fan X., Pan Q., Qin G., Zhang D., Chen L., Zhou J. (2020). Calcitonin gene-related peptide facilitates sensitization of the vestibular nucleus in a rat model of chronic migraine. J. Headache Pain.

[14] Tian R., Zhang Y., Pan Q., Wang Y., Wen Q., Fan X., Qin G., Zhang D., Chen L., Zhang Y. (2022). Calcitonin gene-related peptide receptor antagonist BIBN4096BS regulates synaptic transmission in the vestibular nucleus and improves vestibular function via PKC/ERK/CREB pathway in an experimental chronic migraine rat model. J. Headache Pain.

[15] Bullitt E. (1990). Expression of c-fos-like protein as a marker for neuronal activity following noxious stimulation in the rat. J. Comp. Neurol..

[16] Rahman S.M., Hauser C., Faucher S., Fine E., Luebke A.E. (2023). Both systemic Calcitonin Gene Related Peptide (CGRP) and a vestibular challenge promote anxiety-related behaviors and dynamic imbalance in mice. bioRxiv.

[17] Russo A.F. (2015). Calcitonin gene-related peptide (CGRP): A new target for migraine. Annu. Rev. Pharmacol. Toxicol..

[18] Garland S.G., Smith S.M., Gums J.G. (2019). Erenumab: A First-in-Class Monoclonal Antibody for Migraine Prevention. Ann. Pharmacother..

[19] Wang X., Chen Y., Song J., You C. (2021). Efficacy and safety of monoclonal antibody against calcitonin gene-related peptide or its receptor for migraine: A systematic review and network meta-analysis. Front. Pharmacol..

[20] Drellia K., Kokoti L., Deligianni C.I., Papadopoulos D., Mitsikostas D.D. (2021). AntiCGRP monoclonal antibodies for migraine prevention: A systematic review and likelihood to help or harm analysis. Cephalalgia.

[21] Vandervorst F., Van Deun L., Van Dycke A., Paemeleire K., Reuter U., Schoenen J., Versijpt J. (2021). CGRP monoclonal antibodies in migraine: An efficacy and tolerability comparison with standard prophylactic drugs. J. Headache Pain.

[22] Torres-Ferrus M., Alpuente A., Pozo-Rosich P. (2019). How much do calcitonin gene-related peptide monoclonal antibodies improve the quality of life in migraine? A patient’s perspective. Curr. Opin. Neurol..

[23] Hoskin J.L., Fife T.D. (2022). New Anti-CGRP Medications in the Treatment of Vestibular Migraine. Front. Neurol..

[24] Barbanti P., Egeo G., Aurilia C., Altamura C., d’Onofrio F., Finocchi C., Albanese M., Aguggia M., Rao R., Zucco M. (2022). Predictors of response to anti-CGRP monoclonal antibodies: A 24-week, multicenter, prospective study on 864 migraine patients. J. Headache Pain.

[25] Lovato A., Marioni G., Monzani D., Rossettini G., Genovese E., de Filippis C. (2021). Physical Therapy for Benign Positional Vertigo of Posterior Canal: The Role of Alternated Epley and Semont Maneuvers. Ear Nose Throat J..

[26] Lovato A., Frosolini A., Marioni G., de Filippis C. (2021). Higher incidence of Ménière’s disease during COVID-19 pandemic: A preliminary report. Acta Otolaryngol..

[27] Iglebekk W., Tjell C. (2022). High score of dizziness-handicap-inventory (DHI) in patients with chronic musculoskeletal pain makes a chronic vestibular disorder probable. Scand J. Pain..

[28] Molnár A., Maihoub S., Mavrogeni P., Tamás L., Szirmai Á. (2022). Depression scores and quality of life of vertiginous patients, suffering from different vestibular disorders. Eur. Arch. Otorhinolaryngol..

[29] Balci B., Akdal G. (2022). Outcome of vestibular rehabilitation in vestibular migraine. J. Neurol..

[30] Fu W., Wang Y., He F., Wei D., Bai Y., Han J., Wang X. (2021). Vestibular and oculomotor function in patients with vestibular migraine. Am. J. Otolaryngol..

[31] Salviz M., Yuce T., Acar H., Karatas A., Acikalin R.M. (2016). Propranolol and venlafaxine for vestibular migraine prophylaxis: A randomized controlled trial. Laryngoscope.

[32] Russo C.V., Saccà F., Braca S., Sansone M., Miele A., Stornaiuolo A., De Simone R. (2023). Anti-calcitonin gene-related peptide monoclonal antibodies for the treatment of vestibular migraine: A prospective observational cohort study. Cephalalgia.

[33] Bayer O., Adrion C., Al Tawil A., Mansmann U., Strupp M., PROVEMIG Investigators (2019). Results and lessons learnt from a randomized controlled trial: Prophylactic treatment of vestibular migraine with metoprolol (PROVEMIG). Trials.

[34] Lepcha A., Amalanathan S., Augustine A.M., Tyagi A.K., Balraj A. (2014). Flunarizine in the prophylaxis of migrainous vertigo. Eur. Arch. Otolaryngol..

[35] Yiannakis C., Hamilton L., Slim M., Kontorinis G. (2022). A systematic review and meta-analysis of prophylactic medication of vestibular migraine. J. Laryngol. Otol..

[36] Maldonado Fernández M., Birdi J.S., Irving G.J., Murdin L., Kivekäs I., Strupp M. (2015). Pharmacological agents for the prevention of vestibular migraine. Cochrane Database Syst. Rev..

[37] Smyth D., Britton Z., Murdin L., Arshad Q., Kaski D. (2022). Vestibular migraine treatment: A comprehensive practical review. Brain A J. Neurol..

